# Is there a role for carbohydrate restriction in the treatment and prevention of cancer?

**DOI:** 10.1186/1743-7075-8-75

**Published:** 2011-10-26

**Authors:** Rainer J Klement, Ulrike Kämmerer

**Affiliations:** 1 Department of Radiation Oncology, University hospital of Würzburg, D-97080 Würzburg, Germany; 2 Department of Obstetrics and Gynaecology, University hospital of Würzburg, D-97080 Würzburg, Germany

**Keywords:** Ketogenic diet, cancer, review, low carbohydrate diet, cachexia, insulin, insulin-like growth factor 1 (IGF1)

## Abstract

Over the last years, evidence has accumulated suggesting that by systematically reducing the amount of dietary carbohydrates (CHOs) one could suppress, or at least delay, the emergence of cancer, and that proliferation of already existing tumor cells could be slowed down. This hypothesis is supported by the association between modern chronic diseases like the metabolic syndrome and the risk of developing or dying from cancer. CHOs or glucose, to which more complex carbohydrates are ultimately digested, can have direct and indirect effects on tumor cell proliferation: first, contrary to normal cells, most malignant cells depend on steady glucose availability in the blood for their energy and biomass generating demands and are not able to metabolize significant amounts of fatty acids or ketone bodies due to mitochondrial dysfunction. Second, high insulin and insulin-like growth factor (IGF)-1 levels resulting from chronic ingestion of CHO-rich Western diet meals, can directly promote tumor cell proliferation via the insulin/IGF1 signaling pathway. Third, ketone bodies that are elevated when insulin and blood glucose levels are low, have been found to negatively affect proliferation of different malignant cells *in vitro *or not to be usable by tumor cells for metabolic demands, and a multitude of mouse models have shown anti-tumorigenic properties of very low CHO ketogenic diets. In addition, many cancer patients exhibit an altered glucose metabolism characterized by insulin resistance and may profit from an increased protein and fat intake.

In this review, we address the possible beneficial effects of low CHO diets on cancer prevention and treatment. Emphasis will be placed on the role of insulin and IGF1 signaling in tumorigenesis as well as altered dietary needs of cancer patients.

## Introduction

When defining the factors of a healthy lifestyle that aims at preventing a disease like cancer, a logical approach is to compare individuals that get the disease with those that don't. Cancer, which might be considered a disease of civilization, has consistently been reported to be very rare among uncivilized hunter-gatherer societies [1-4]. This observation makes sense from an evolutionary perspective from which it is reasonable to assume that the lifestyle factors that protect our genome against tumorigenesis have been selected for early in the history of the genus *homo *when humans lived as hunter-gatherers [5]. In particular, the time since the neolithic revolution, which meant the transition from foraging and nomadism to agriculture and settlement, spans a fraction less than 1% of human history. Thus, the switch from the "caveman's diet" consisting of fat, meat and only occasionally roots, berries and other sources of carbohydrate (CHO) to a nutrition dominated by easily digestible CHOs derived mainly from grains as staple food would have occurred too recently to induce major adoptions in our genes encoding the metabolic pathways. This is even more the case for the changes that occurred over the past 100 years, in particular the switch from labor in the field to a sedentary lifestyle and an increase in the consumption of easily digestible CHOs with high glycemic indices (GIs), leading to diseases of civilization that are strongly associated with the so-called Western way of life [6]. Despite a large heterogeneity in regional occupation, modern hunter-gatherers share certain lifestyle factors that are not frequently met in Westernized societies, including regular physical activity, sun exposure, sufficient sleep, low chronic stress and the lack of foods that would also not have been available to our pre-neolithic ancestors. While there is already compelling evidence for the beneficial roles of regular physical activity and sufficient vitamin D in the prevention and treatment of cancer, the influence of the altered nutritional patterns in the Western diet is less clearly defined.

### Modern hunter-gatherers' diet

Data from 229 hunter-gatherer societies included in the revised *Ethnographic Atlas *indicate that hunter-gatherer diets differ from typical Western ones in basically two aspects: first, a strong reliance on animal foods (45-65% of energy or E%) and second, the consumption of low-GI plant foods such as vegetables, fruits, seeds and nuts [7]. This is consistent with stable isotope studies of human fossils [8,9]. As a consequence, the amount and type of carbohydrates in the typical western diet differ markedly from the ones that our genes adapted to. In particular, Cordain and colleagues estimated that modern hunter-gatherers derived about 22-40 E% from CHOs and 19-30 E% from protein, which is lower and higher, respectively, than recommended by Western food agencies. Recently, Ströhle & Hahn confirmed that the energy derived from CHOs - despite being dependent upon geographic latitude and ecological environment - in modern hunter-gatherers is markedly lower than in Westernized societies [10]. High CHO intake, in particular in the form of sugar and other high GI foods, has been linked to modern diseases like metabolic syndrome [11], Alzheimer's disease [12,13], cataract and macula degeneration [14-16] and gout [17]. Intriguingly, with the possible exception of Alzheimer's disease [18], the occurrence and prognosis of cancer seems positively associated with both the prevalence of these diseases [19-28] and the GI and glycemic load (GL) of the diet [29-32]; this implies a possible role of high CHO intake in cancer as well.

In this review, we are going to present some arguments that support the hypothesis that lowering the amount of CHOs in the diet can have direct beneficial effects on the prevention and treatment of malignant diseases. The main focus will be on very low CHO, ketogenic diets as an effective supportive therapy option for cancer patients.

## Tumor cell metabolism - it's all about glucose

That there exists an intimate connection between CHOs and cancer has been known since the seminal studies performed by different physiologists in the 1920s. Treating diabetic patients, A. Braunstein observed in 1921 that in those who developed cancer, glucose secretion in the urine disappeared. Further, culturing tissue of benign and malign origin in glucose-containing solutions, he quantified the much higher consumption by cancer tissue compared to muscle and liver [33]. One year later, R. Bierich described the remarkable accumulation of lactate in the micromilieu of tumor tissues [34] and demonstrated lactate to be essential for invasion of melanoma cells into the surrounding tissue [35]. The most accurate and well known experiments were published by Otto Warburg and colleagues from 1923 on [36-38]. Warburg observed that tumor tissue *ex vivo *would convert high amounts of glucose to lactate even in the presence of oxygen (aerobic glycolysis), a metabolic phenotype now referred to as the Warburg effect. This meant a sharp contrast to normal tissue which was known to exhibit the Pasteur effect, i.e., a decrease of glucose uptake and inhibition of lactate production under aerobic conditions. Today, the Warburg effect is an established hallmark of cancer, i.e., a pathological capability common to most, if not all, cancer cells [39]. At first sight, the reason why many cancers should run preferentially on glucose to produce energy seems counter-intuitive: basic biochemistry textbooks tell us that glycolysis partially oxidizes the carbon skeleton of one mole of glucose to two moles of pyruvate, yielding two moles of ATP and NADH. In normal cells under normoxic conditions, pyruvate is oxidized in the mitochondria by the enzyme pyruvate dehydrogenase, creating acetyl-CoA which is further utilized in the tricarboxylic acid cycle (TCA or Krebs cycle) to yield a total of 32+ moles of ATP. Thus, the oxidation of pyruvate in the mitochondria supplies 30+ additional moles of ATP compared to its reduction to lactate via lactate dehydrogenase A (LDHA), which happens in case of insufficient oxygen levels or - in case of cancer cells - due to the Warburg effect.

### Possible causes for the "Warburg effect"

**Figure 1:**
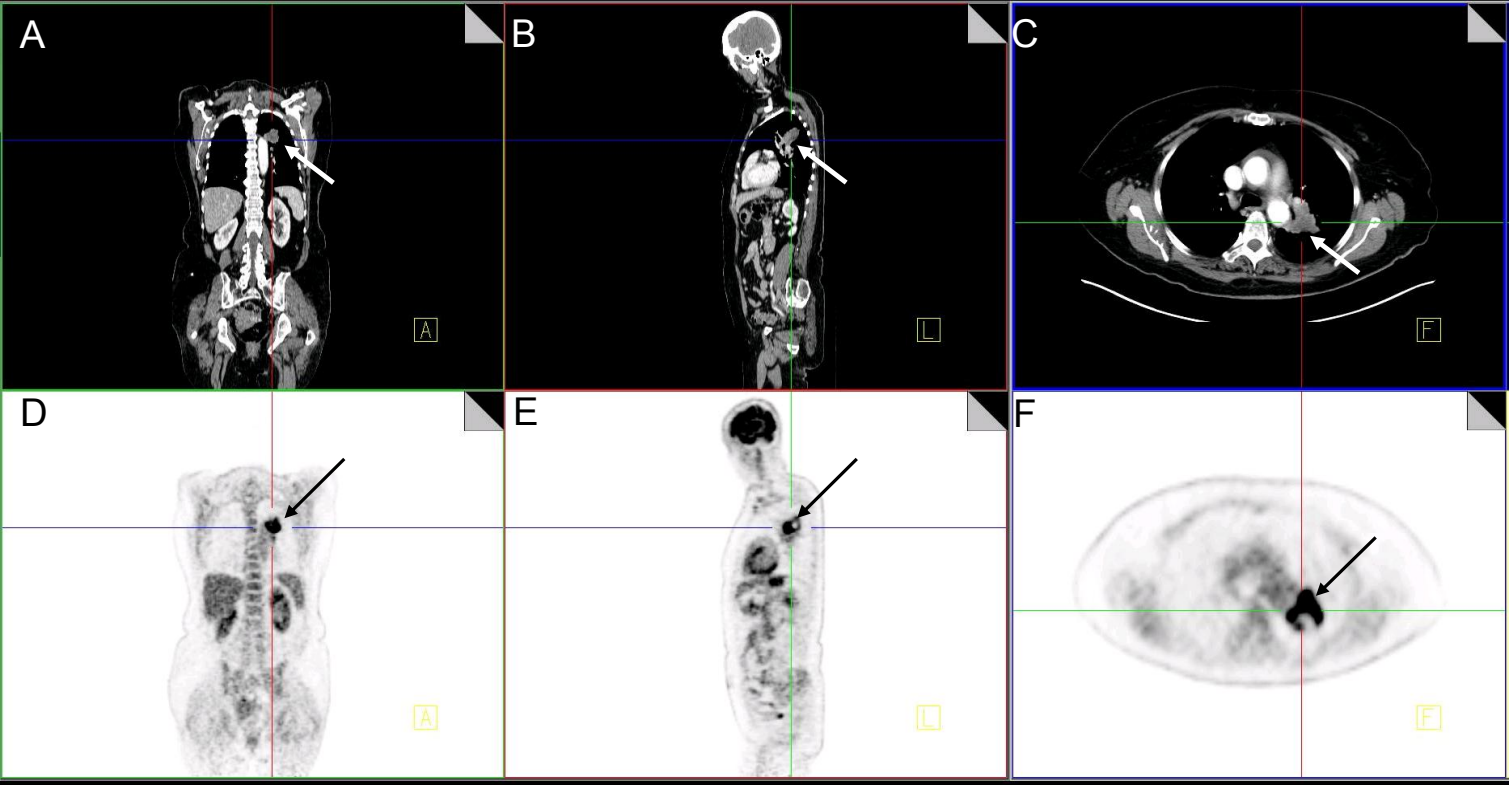
**PET image of a patient with a left central lung carcinoma (arrows)**. Note also the high FDG uptake by the kidneys (Fig D), brain and myocard (Figure E). Source: PET/CT Imaging Centre, University Hospital of Würzburg.

Over the past years, however, it has become increasingly clear that malignant cells compensate for this energy deficit by up-regulating the expression of key glycolytic enzymes as well as the glucose transporters GLUT1 and GLUT3, which have a high affinity for glucose and ensure high glycolytic flux even for low extracellular glucose concentrations. This characteristic is the basis for the wide-spread use of the functional imaging modality positron emission tomography (PET) with the glucose-analogue tracer ^18^F-fluoro-2-deoxyD-glucose (FDG) (Figure 1). There are mainly four possible drivers discussed in the literature that cause the metabolic switch from oxidative phosphorylation to aerobic glycolysis in cancer cells. The first one is mitochondrial damage or dysfunction [40], which was already proposed by Warburg himself as the cause for tumorigenesis [41]. Somatic mutations in mitochondrial DNA (mtDNA) and certain OXPHOS genes can lead to increased production of reactive oxygen species (ROS) and accumulation of TCA cycle intermediates (succinate and fumarate) that trigger the stabilization of hypoxia inducible factor (HIF)-1α, inactivation of tumor suppressors including p53 and PTEN and upregulation of several oncogenes of the phosphoinositide 3-kinase (PI3K)/Akt/mammalian target of rapamycin (mTOR) signaling pathway [42]. In tumor cells, Akt plays a major role in resisting apoptosis and promoting proliferation, and it does so by reprogramming tumor cell metabolism [43-45]. Akt suppresses β-oxidation of fatty acids [46], but enhances *de novo *lipid synthesis in the cytosol [47,48]. Akt also activates mTOR, a key regulator of cell growth and proliferation that integrates signaling from insulin and growth factors, amino acid availability, cellular energy status and oxygen levels [49,50]. In cancer cells, mTOR has been shown to induce aerobic glycolysis by up-regulating key glycolytic enzymes, in particular through its downstream effectors c-Myc and HIF-1α. Both of these transcription factors are involved in the expression of pyruvate kinase M2, a crucial glycolytic enzyme for rapidly proliferating cells [51].

**Figure 2:**
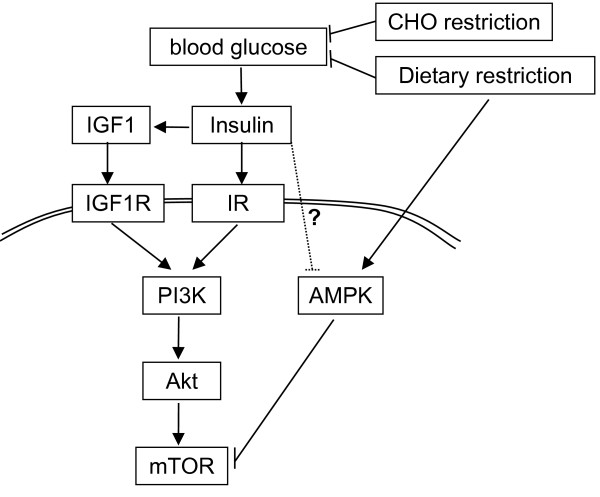
**The IGF1R-IR/PI3K/Akt/mTOR pathway and its manipulation through diet**. Elevations in blood glucose concentrations lead to secretion of insulin with subsequent elevation of free IGF1. Binding of insulin and IGF1 to their receptor tyrosine kinases induces autophosphorylation of the latter which leads to subsequent activation of PI3K by one of at least three different pathways [54]. Further downstream, PI3K signaling causes phosphorylation and activation of the serine/threonine kinase Akt (also known as protein kinase B). Akt activates mammalian target of rapamycin (mTOR), which itself induces aerobic glycolysis by up-regulating key glycolytic enzymes, in particular via its downstream effectors c-Myc and hypoxia inducible factor (HIF)-1α. mTOR is negatively affected through activation of AMPK, which can be achieved by dietary restriction [67]. In addition, a possible negative interaction between insulin and AMPK is discussed *in vivo *[60].

HIF-1α is further important for the adaption to hypoxia by increasing the expression of glycolytic enzymes including GLUT1 and hexokinase (HK)II as well as several angiogenic factors [49,52]. The observation that certain malignant cells are able to use both glycolysis and OXPHOS under aerobic conditions has been taken to argue that mitochondrial dysfunction alone is not a sufficient cause for the Warburg effect [53]. Indeed, somatic mutations in most oncogenes and tumor suppressor genes have been shown to directly or indirectly activate glycolysis even in the presence of oxygen. As described above, they do so mainly by hyperactivating major metabolic signaling pathways such as the insulin-like growth facor-1 receptor (IGFR1)-insulin receptor (IR)/PI3K/Akt/mTOR signaling pathway (Figure 2). In principle, hyperactivation of this pathway can occur at several points from alterations in either upstream (receptor) or downstream (transducer) proteins and/or disruption of negative feedback loops via loss-of-function mutations in suppressor genes [44,45,54]. Thus, genetic alterations in oncogenes and tumor suppressor genes are a second possible cause for the Warburg effect.

As a third mechanism, with advanced tumorigenesis, non-mutation induced stabilization of HIF-1α occurs through a lack of oxygen in hypoxic tumor regions and contributes to increased glycolysis. Proliferation of aggressive tumors proceeds too fast for concurrent vascularization, so that hypoxic regions will develop. Because the diffusion coefficients for glucose are larger than for oxygen, these regions rely heavily on glycolysis. Hypoxic cancer cells are particularly radio- and chemoresistant. In PET-studies, tumor areas with high FDG uptake have been consistently linked to poor prognosis [55,56] and are now being considered as important biological target volumes to receive dose escalations in radiation treatment [57].

### The impact of insulin and IGF1

Finally, chronic activation of the IGFR1-IR/PI3K/Akt survival pathway through high blood glucose, insulin and inflammatory cytokines has been proposed as a cause of carcinogenesis [30,58,59] and switch towards aerobic glycolysis. In this theory, hyperactivation of the IGFR1-IR signalling pathway does not occur primarily through somatic gene mutations, but rather through elevated concentrations of insulin and IGF1, allowing for more ligands binding to their receptors. Interestingly, gain-of-function mutations resulting in ligand-independent overactivation of both IGFR1 and IR are uncommon [60]. Furthermore, loss-of-function of the tumor suppressor PTEN may result in hypersensitivity to insulin/IGF1-mediated activation of the IGFR1-IR pathway rather than constitutive downstream activation [60]. Thus, it seems possible that high levels of insulin and IGF1 in the microenvironment favor cell survival and evolution towards malignancy instead of apoptosis in DNA-damaged cells. Indeed, both hyperglycemia and hyperinsulinemia are predictors of cancer occurrence and cancer-related mortality [23,25,26]. This highlights the link between the metabolic syndrome and cancer on the one hand and cancer and lifestyle factors like nutrition on the other. As indicated in Figure 2, restriction of dietary CHOs would counteract this signalling cascade by normalizing glucose and insulin levels in subjects with metabolic syndrome, in this way acting similar to calorie restriction/fasting [61,62]. Indeed, it has been shown in healthy subjects that CHO restriction induces hormonal and metabolic adaptions very similar to fasting [63-66]. Dietary restriction is able to inhibit mTOR signalling through a second, energy-sensing pathway by stimulating phosphorylation of AMP-activated protein kinase (AMPK) [67]. *In vitro*, AMPK phosphorylation is sensitive to the ratio of AMP/ATP within the cell; *in vivo*, however, concentrations of glucose and other nutrients are kept fairly stable throughout calorie restriction, suggesting that hormones such as insulin and glucagon might play a more dominant role in regulating AMPK and thus mTOR activation [60]. This may open a second route to mimic the positive effects of calorie restriction through CHO restriction (Figure 2).

### Glycolysis: beneficial for tumor cells

Besides the ability to grow in hypoxic environments, a high glycolytic rate has several additional advantages for the malignant cell: First, it avoids the production of ROS through OXPHOS. Second, the phosphometabolites that accumulate during glycolysis can be processed in the pentose phosphate pathway for biosynthesis of nucleic acids and lipids. Similarly, overexpession of Akt induces an increased flow of pyruvate-derived citrate from the mitochondrion into the cytosol, where it is used for lipid biosynthesis. Third, a tumor cell focusing on glycolysis no longer relies on intact mitochondria and may evade apoptotic signalling which is linked to mitochondrial function. In addition, the genes and pathways that up-regulate glycolysis are themselves anti-apoptotic [40]. Fourth, high glycolytic activity produces high levels of lactate and H^+ ^ions which get transported outside the cell where they directly promote tumor aggressiveness [68] through invasion and metastasis, two other hallmarks of cancer. For this purpose, glycolytic tumor cells often show overexpression of monocarboxylate transporters (MCTs) and/or Na^+^/H^+ ^exchangers [69] that allow them to effectively remove large amounts of H^+ ^ions. For MDA-MB-231 breast cancer cells it has been shown that lactate drives migration by acting as a chemo-attractant and enhances the number of lung metastasis in athymic nude mice [70]. Lactate can also be taken up and used as a fuel by some malignant cells, and oxidative tumor cells have been shown to co-exist with glycolytic ones (both stromal and malignant) in a symbiotic fashion [71]. In glioma cells, lactate upregulates and activates the matrix metalloproteinase (MMP)-2 which degrades the extra-cellular matrix and basement membrane [72]. Activation of MMPs may also occur in the microenvironment through low pH values in a similar way as discussed for carious decay of the dentin organic matrix through lactate released by cariogenic bacteria [73]. Acidification of the microenvironment further induces apoptosis in normal parenchymal and stromal cells [74,75] and therefore provides a strong selective growth advantage for tumor cells that are resistant to low pH-induced apoptosis [76,77].

## Glucose availability as a promoter of cancer growth

Taken together, increased glucose flux and metabolism promotes several hallmarks of cancer such as excessive proliferation, anti-apoptotic signalling, cell cycle progression and angiogenesis. It does so, however, at the expense of substrate inflexibility compared to normal cells. It is clear that the high proliferative phenotype can only be sustained as long as a steady supply of substrates for ATP production is available. Thus, with progressive tumorigenesis, cancer cells become more and more 'addicted' to aerobic glycolysis [53] and vulnerable to glucose deprivation. Indeed, several studies have shown that malignant cells *in vitro *quickly lose ATP and commit apoptosis when starved of glucose [78-80]. Masur et al. showed that diabetogenic glucose concentrations (11 mM) compared to physiological ones (5.5 mM) lead to altered expression of genes that promote cell proliferation, migration and adhesion in tumor cell lines from several organs including breast, colon, prostate and bladder [81]. Adding insulin to the high-glucose medium further enhanced proliferation rates by 20-40% and promoted activation of the PI3K pathway. The question is whether altered blood glucose levels have similar effects on tumor growth *in vivo*. Theoretically, low blood glucose might cut some of the most hypoxic tumor cells from their diffusion-limited fuel supply. Gatenby and Gillies originally proposed this mechanism as an explanation for necrotic areas often found within tumor tissue [82], but they later revised this hypothesis based on a mathematical model that predicted only a modest decline of glucose concentrations with distance from the closest blood vessel [69]. There are, however, several lines of evidence pointing towards a strong correlation between blood glucose levels and tumor growth *in vivo *that might indicate other important effects mediated by glucose. For example, the reduction of plasma glucose levels in tumor-bearing animals induced through calorie restriction may be responsible, directly or indirectly, for the significantly prolonged survival compared to normal-fed controls [83,84]. In 1962, Koroljow reported the successful treatment of two patients with metastatic tumors by an insulin-induced hypoglycemic coma [85]. Hyperglycemia, on the other hand, is a predictor of poor survival in patients with various cancers [22,26,86-88] and has been positively correlated to an increased risk for developing cancer at several sites including the pancreas, esophagus, liver, colon, rectum, stomach and prostate in large cohort studies [25,89,90].

### Indirect effects of glucose availability

Besides delivering more glucose to the tumor tissue, hyperglycemia has two other important negative effects for the host: First, as pointed out by Ely and Krone, even modest blood glucose elevations as they typically occur after a Western diet meal competitively impair the transport of ascorbic acid into immune cells [88,91]. Ascorbic acid is needed for effective phagocytosis and mitosis, so that the immune response to malignant cells is diminished. Second, it has been shown *in vitro *and *in vivo *that hyperglycemia activates monocytes and macrophages to produce inflammatory cytokines that play an important role also for the progression of cancer [92-94] (see below). Third, high plasma glucose concentrations elevate the levels of circulating insulin and free IGF1, two potent anti-apoptotic and growth factors for most cancer cells [60]. Free IGF1 is elevated due to a decreased transcription of IGF binding protein (IGFBP)-1 in the liver mediated by insulin [95]. Due to expression of GLUT2, the β-cells of the pancreas are very sensitive to blood glucose concentration and steeply increase their insulin secretion when the latter exceeds the normal level of ~5 mM. In the typical Western diet consisting of three meals a day (plus the occasional CHO-rich snacks and drinks), this implies that insulin levels are elevated above the fasting baseline over most of the day. Both insulin and IGF1 activate the PI3K/Akt/mTOR/HIF-1α pathway by binding to the IGF1 receptor (IGF1R) and insulin receptor (IR), respectively (Figure 2). In addition, insulin stimulates the release of the pro-inflammatory cytokine interleukin (IL)-6 from human adipocytes [96]. Thus, it could be hypothesized that a diet which repeatedly elevates blood glucose levels due to a high GL provides additional growth stimuli for neoplastic cells. In this respect, Venkateswaran et al. have shown in a xenograft model of human prostate cancer that a diet high in CHO stimulated the expression of IRs and phosphorylation of Akt in tumor tissue compared to a low CHO diet [97]. In colorectal [27], prostate [24] and early stage breast cancer patients [23,98] high insulin and low IGFBP-1 levels have been associated with poor prognosis. These findings again underline the importance of controlling blood sugar and hence insulin levels in cancer patients. Dietary restriction and/or a reduced CHO intake are straightforward strategies to achieve this goal.

## Altered nutritional needs of cancer patients

**Figure 3:**
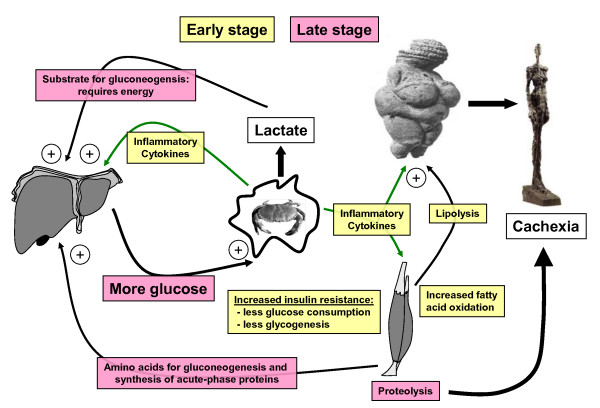
**Development of the cachectic state via sustained inflammatory signaling**. Glucose metabolism in peripheral tissues is impaired already at early stages, while hepatic gluconeogenesis increases during tumor progression at later stages.

Cancer patients and those with metabolic syndrome share common pathological abnormalities. Since 1885, when Ernst Freund described signs of hyperglycemia in 70 out of 70 cancer patients [99], it has been repeatedly reported that glucose tolerance and insulin sensitivity are diminished in cancer patients even before signs of cachexia (weight loss) become evident [100-102]. Both diabetes and cancer are characterized by a common pathophysiological state of chronic inflammatory signalling and associated insulin resistance. In cancer patients, insulin resistance is thought to be mediated by an acute phase response that is triggered by pro-inflammatory cytokines such as tumor necrosis factor (TNF)-α [101] and IL-6 [103]. In animal and human studies, removal of the tumor resulted in improved glucose clearance, suggesting that these cytokines are secreted, at least in part, from the tumor tissue itself [104,105]. The impact on the metabolism of the host is illustrated in Figure 3. In the liver, the inflammatory process leads to increased gluconeogenesis that is fuelled by lactate secreted from the tumor as well as glycerol from fatty acid breakdown and the amino acid alanine [106] from muscle proteolysis. Gluconeogenesis is an energy-consuming process and might contribute to cancer cachexia by increasing total energy expenditure. Despite increased lipolysis, hepatic production of ketone bodies is usually not enhanced in cancer patients [107,108]. This is in contrast to starvation, where the ketone bodies acetoacetate and β-hydroxybutyrate counteract proteolysis by providing energy for the brain and muscles [109]. In muscle, glucose uptake and glycogen synthesis are inhibited already at early stages of tumor progression, while fatty acid oxidation remains at normal levels or is increased [110,111]. In the latter case, more fat has to be provided from lipolysis in the adipose tissue. In addition, muscles progressively lose protein to provide amino acids for hepatic synthesis of acute-phase proteins and as precursors for gluconeogenesis. Thus, insulin resistance contributes to fat loss and muscle wasting, the two hallmarks of cancer cachexia. At the same time, it makes more glucose in the blood available for tumor cells.

### Fat and ketone bodies: anti-cachectic effects

It therefore seems reasonable to assume that dietary carbohydrates mainly fuel malignant cells which express the insulin-independent glucose transporters GLUT1 and GLUT3, while muscle cells are more likely to benefit from an increased fat and protein intake. This was summarized as early as in 1977 by C. Young, who stated that lipid sources predominate the fuel utilization of peripheral tissue of patients with neoplastic disease compared to healthy subjects [112]. In addition, most malignant cells lack key mitochondrial enzymes necessary for conversion of ketone bodies and fatty acids to ATP [40,113,114], while myocytes retain this ability even in the cachectic state [107]. This led some authors to propose a high-fat, ketogenic diet (KD) as a strategy to selectively improve body composition of the host at the expense of the tumor [113,115,116]. The traditional KDs, which recommended protein and CHO to account, in combination, for roughly 20 E% (in the incorrect assumption that they were equivalent due to gluconeogenesis) and fat for the remaining 80 E%, have been widely used to treat childhood epilepsia since the 1920s [117]. KDs are also used to treat adiposity [118] and currently adult epilepsy [119]. In the 1980s, Tisdale and colleagues investigated the effects of a ketogenic diet consisting mainly of medium chain triglycerides (MCTs) on two aggressive animal tumor models that were known to lack the ability to utilize ketone bodies. While the diet had no effect on rats bearing the Walker 256 sarcoma [120], it decreased the cachectic weight loss in proportion to its fat content in mice bearing the mouse-specific colon carcinoma MAC16 [121]. For the latter, they further proved an anti-cachectic effect of a ketogenic diet in which the MCTs were replaced with long chain triglycerides (LCTs), although to a somewhat lesser extent [122]. Contrary to LCTs, MCTs do not require transport in chylomicrones, but readily reach the liver where they are metabolized to yield high amounts of ketone bodies. Interestingly, administration of insulin was able to reduce the weight loss similar to the ketogenic MCT diet, but at the expense of a 50% increase in tumor size, which could be counteracted by addition of β-hydroxybutyrate in the drinking water [123]. The supporting effect of insulin on tumor growth has been known since 1924, when Händel and Tadenuma described the nourishing effect of insulin on tumor tissue in an animal model [124], showing evidence that reducing insulin might reduce tumor growth.

### Clinical studies on fat and cachexia

Clinical studies investigating the anti-cachectic effects of high-fat diets are, however, rare. Fearon et al. administered a 70% MCT diet supplemented with β-hydroxybutyrate parenterally to five late-stage cachectic patients. After seven days on the diet, mean body weight had increased by 2 kg and their physical performance status had improved [125]. Nebeling et al. investigated the effects of a MCT-based ketogenic diet taken *ad libitum *(60% MCT oil, 20% protein, 10% CHO, 10% other fats) on body weight and glucose metabolism in two pediatric patients with advanced-stage astrocytoma. Within 7 days on the diet, blood glucose levels had decreased to normal, while glucose uptake by the tumor estimated from FDG-PET scans had decreased by an average value of 21.8%. Notably, body weight remained stable throughout the study period of 8 weeks. In a randomized controlled study, Breitkreuz et al. showed that by supplementing the normal diet of 11 under-nourished, non-diabetic patients suffering from metastatic gastro-intestinal cancers with a fat-enriched liquid supplementation for 8 weeks, it was possible to reverse the loss of body weight and lean tissue mass and to improve several quality-of-life parameters in the treatment group, while the control group continued to lose body and lean tissue weight [126]. The supplement contained 66% energy from fat, of which 45% were monounsaturated, 27% saturated (both LCT and MCT) and 28% polyunsaturated; mean energy intake ranged between 1000 and 2000 kcal/day and tended to be higher in patients receiving the additional fat drink.

## The benefits of mild ketosis

The study of Breitkreuz et al. shows that ketosis might not be necessary to improve the cachectic state of cancer patients. In recent years, however, more evidence has emerged from both animal and laboratory studies indicating that cancer patients could benefit further from a very low CHO KD. In their mouse models, Tisdale et al. already noted that the KD not only attenuated the cachectic effects of the tumor, but also that the tumors grew more slowly (although they did not attribute this to a direct anti-tumor effect of β-hydroxybutyrate). Tumor growth inhibition through a KD has now been established in many animal models, is supported by a few clinical case reports, and laboratory studies have begun to reveal the underlying molecular mechanisms.

### In vitro studies

More than 30 years ago, Magee et al. were the first to show that treating transformed cells with various, albeit supra-physiological, concentrations of β-hydroxybutyrate causes a dose-dependent and reversible inhibition of cell proliferation [116]. Their interpretation of the results that ''...ketone bodies interfere with either glucose entry or glucose metabolism...'' has been confirmed and further specified by Fine et al., who connected the inhibition of glycolysis in the presence of abundant ketone bodies to the overexpression of uncoupling protein-2 (UCP-2), a mitochondrial defect occurring in many tumor cells [127]. In normal cells, abundant acetyl-CoA and citrate from the breakdown of fatty acids and ketone bodies would inhibit key enzymes of glycolysis to ensure stable ATP levels; in tumor cells, however, the same phenomenon would imply a decrease in ATP production if the compensatory ATP production in the mitochondria was impaired. For several colon and breast cancer cell lines, Fine et al. showed that the amount of ATP loss under treatment with acetoacetate was related to the level of UCP-2 expression.

Very recently, Maurer et al. demonstrated that glioma cells - although not negatively influenced by β-hydroxybutyrate - are not able to use this ketone body as a substitute for glucose when starved of the latter, contrary to benign neuronal cells [128]. This supports the hypothesis that under low glucose concentrations, ketone bodies could serve benign cells as a substitute for metabolic demands while offering no such benefit to malign cells.

### Animal studies

To our knowledge, the first and - with a total of 303 rats and nine experiments - most extensive study of a KD in animals was conducted by van Ness van Alstyne and Beebe in 1913 [129]. Experiments were divided into two classes: in the first class, rats in the treatment arm were fed a CHO-free diet consisting of casein and lard for several weeks before plantation of a Buffalo sarcoma, while the control arm received either bread only or casein, lard and lactose. Rats on the CHO-free diet not only gained more weight than the controls, but also exhibited much less tumor growth and mortality rates, the differences being "... so striking as to leave no room for doubt that the diet was an important factor in enabling the rats to resist the tumor after growth had started." In a second class of experiments using either the slow-growing Jensen sarcoma or the aggressive Buffalo sarcoma, the rats were put on the CHO-free diet on the same day that the tumor was planted. This time, differences between the treatment and control groups were "... so slight that ... one is left in no doubt of the ineffectiveness of non-carbohydrate feeding begun at the time of tumor implantation." Interestingly, this parallels the observation of Fearon et al. that rats who started to receive a KD at the same day as tumor transplantation did not differ from controls in either body or tumor weight after 14 d [120]. In these rats, it was noted that despite persistent ketosis, blood glucose levels were not significantly lower than in controls which were also fed *ad libitum*. This stability of blood glucose, independent of ketosis, was subsequently confirmed in studies in which mice were fed *ad libitum *on a KD [84,114,121-123,130] although two studies reported a drop in blood glucose concentrations compared with the control group [116,131]. In the study of Magee et al., however, diet was presented as a liquid vegetable oil and energy intake was not monitored, allowing for the possibility that the animals underate voluntarily, in this way consuming a "caloric restricted KD" used in several experimental settings from the Seyfried lab [84,114,132], which was shown therein to be superior to the unrestricted KD in tumor growth control. That "caloric restriction" *per se *can hamper tumor growth has been impressively demonstrated already in 1942 by A. Tannenbaum in a series of comprehensive mouse models with different mouse strains and tumor induction types [133]. Throughout all experimental series, a strict restriction of food intake (impeding weight gain) several weeks before inducing tumorigenesis by application of 3,4 benzpyrene decreased the appearance rate and appearance time of tumors in the diet mice compared to the *ad libitum *controls. Notably, the calorie-restricted diet was composed of 53% CHOs compared to 69% in the control group. Despite a lack of data on blood glucose and ketone body levels, it could be speculated that the strict restriction of food *per se *(to 50-60% of the control group) induced a ketotic state and thus the ketones were - at least to some extend - responsible for the effects observed.

In Table 1, we summarize the main results of various mouse studies that determined the effects of KDs on tumor growth and host survival. The results seem to indicate an anti-tumor effect of ketosis. Freedland et al. indeed reported that the mice with the highest levels of ketone bodies had the longest survival times in a human prostate cancer xenograft model [134]. But other studies suggest that there are further possible factors to consider. Seyfried et al. used linear regression to show that plasma glucose and IGF1 levels are a better predictor of tumor growth than ketone bodies in a murine astrocytoma model [84]. Tumor growth in this as well as in a follow-up [114] study was only retarded when the KD had been restricted to induce body weight loss, again underlining the effect of caloric restriction *per se*. This contrasts with other studies showing growth-inhibitory effects of unrestricted or higher-caloric KDs despite neither decreases in blood glucose concentration nor body weight loss compared with a control group [130,134,135]. According to Otto et al., whose diet had been enriched in MCT and omega-3 fatty acids, fat quality might play a role in explaining these results [130]. The situation in humans might be different as well, as for example Fine et al. found no correlation between calorie intake or weight loss and disease progression in ten patients on an unrestricted KD [136] (see also below).

**Table 1:** Animal studies that have investigated the effects of a KD on tumor progression and host survival

animals	n	tumor	feeding	C/P/F	major fat source	diet initiation	diet duration	BW vs. controls	BG vs. controls	other effects vs. controls	**Ref**.
				**(E%)**		**(d)**	**(d)**				

C57BL/6 mice	18	B16 melanoma	*ad libitum*	0/0/100 ^1^	PUFA vegetable oil	0	14	-	↓ ^b^	lower number of lung metastases ^b^	[116]
BALB/c mice	20	Medina-Oborn-Danielson mammary tumor	restricted to 60 E% of control	30/60/5	hydrogenated vegetable oil	~ 14	70	↓	↓ ^c^	mortality rate ↓ ^c^	[83]
NMR1 mice	> 15	MAC16 colon carcinoma	*ad libitum*	.../.../80 ^2^	MCT emulsion	8	20	↑	-	50% less weight loss ^b^; left35% less tumor weight	[121]
NMR1 mice	...	MAC16 colon carcinoma	*ad libitum*	.../.../80		14 - 21	9	↑	-	36% less weight loss ^a^	[123]
										32% less tumor weight ^c^	
										less nitrogen output ^a^	
C57BL/6 mice	6	CT-2A mouse astrocytoma	restricted to 60 E% of control	0/8/92	lard	1	13	↓ ^3^	↓ ^3^	80% less tumor weight^b^; plasma IGF1 levels ↓ ^b^,^3^	[84]
C57BL/6 mice	11	CT-2A mouse astrocytoma	*ad libitum*	3/17/80	soy oil (KetoCal^©^)	3	> 8	-	-	no significant differences in either tumor weight, survival or vascularity	[114]
+	+	+									
BALB/cJ SCID mice	14	U87 glioblastoma	restricted to 65-70 E% of control	3/17/80	soy oil (KetoCal^©^)	3	>8	↓ ^b^	↓ ^b^	65% (CT-2A)^b^and 35% (U87)^c^less tumor wet weight;	
										longer survival ^b^; lower number of blood vessels (both tumors)	
nu/nu mice	20	LNCaP human prostate cancer	*ad libitum*	10/45/45	...	14	63	↓ ^a^	...	plasma insulin levels ↓ ^c^; plasma IGF1 levels ↓ ^c^;	[97]
										45% less tumor volume ^a^;	
										43% less tumor dry weight^c^;	
										decreased levels of phosphorylated Akt (below detected limits) and insulin receptor in tumor tissue	
SCID mice	25	LAPC-4 human prostate cancer	9% more energy than control	0/16/84	milk fat + lard	-24	> 40	-	↑ ^c^	longer survival ^b^	[134]
NMRI mice	12	23132/87 human gastric adenoma	*ad libitum*	0/14/86	cheese + MCT + omega-3 oil	0	> 16	-	-	longer survival ^a^;	[130]
										tumor growth rate ↓ ^c^;	
										larger necrotic area in tumors ^b^	
C3H/HeN mice ^4^	5	squamous cell car-cinoma VII	*ad libitum*	16/58/26	...	-7	16	↑	↓	41% less tumor volume ^d^	[131]
Foxn1nu mice	12	LNT-229 glioma cells	*ad libitum*	0/13/36	flaxseed and hempseed oil	1	> 63	-	-	no significant differences in survival, tumor growth and plasma IGF1 levels	[128]

In all but one cases, control diets contained a minimum of 40% CHO. Diet initiation refers to the time of tumor cell plantation.

SCID = Severe Combined Immunodeficiency; C/P/F = ratio of CHO:protein:fat; E% = percent of energy; BW = body weight; BG = blood glucose

^1 ^plus not further specified pellets on days 5, 8 and 11/^2 ^plus 3 mg/ml beta-hydroxybutyrate in drinking water/^3 ^controls were fed a KD *ad libitum*, not high-CHO/^4 ^similar results for Rag2M mice bearing human colorectal HCT-116 tumors/^a ^p < 0.005; ^b ^p < 0.01; ^c ^p < 0.05; ^d ^p < 0.1

Concerning fat quality, Freedland et al. observed that a diet rich in corn oil might stimulate prostate cancer growth to a greater extent than one rich in saturated fat [134]. A recent study suggests, however, that tumor growth inhibition neither depends on fat quality nor ketone body levels [131]. In this case, mice injected with either murine squamous cell carcinoma or human colorectal carcinoma cells received a low CHO, high-protein diet in which ~ 60 E% was derived from protein, 10-15 E% from CHO and ~ 25 E% from fat. No systemic ketosis was measured, yet tumors grew significantly less compared with a standard diet containing 55 E% from CHO and 22 E% from the same fat source. IGF1 levels and body weight remained stable, so these findings could not be attributed to one of these factors. There was, however, a significant drop in blood glucose, insulin and lactate levels, and a positive correlation between blood lactate as well as insulin levels and tumor growth was found. The study of Venkateskwaran et al. indicates that in prostate cancer insulin and/or IGF1 play major roles in driving tumor cell proliferation [97].

The diversity of these findings should not be surprising, given the variety of mice strains, tumor cell lines, diet composition and time of diet initiation relative to tumor planting. Instead, it seems remarkable that the same basic treatment, namely drastic restriction of CHOs, apparently induces anti-tumoral effects via different pathways. Thus, it may depend on the circumstances which variables - including blood glucose, insulin, lactate, IGF1, fat quality and ketone bodies - are the best predictors of and responsible for the anti-tumor effects of very low CHO diets.

### Human studies

Until now, no randomized controlled trials have been conducted to evaluate the effects of a KD on tumor growth and patient survival. It has to be noted in general, however, that any dietary intervention requiring a dramatic change of life style makes randomized studies nearly impossible - however, even prospective cohort studies are missing. There is only anecdotal evidence that such a diet might be effective as a supportive treatment. One study investigated whether a high-fat diet (80% non-nitrogenous calories from fat) would inhibit tumor cell replication compared to a high-dextrose diet (100% non-nitrogenous calories from dextrose) in 27 patients with gastro-intestinal cancers [137]. Diets were administered parenterally and cell proliferation assessed using thymidine labeling index on tumor samples. After 14 days, the authors found a non-significant trend for impaired proliferation in the high-fat group. Whether ketosis was achieved with this regime was not evaluated, but blood glucose levels were comparable in both trial groups. A very recent pilot trial demonstrated the feasibility of a low CHO up to a ketogenic regimen implemented for 12 weeks in very advanced outpatient cancer patients. Notably, severe side effects were not observed, nearly all standard blood parameters improved and some measures of quality of life changed for the better [138]. The first attempt to treat cancer patients with a long-term controlled KD was reported by L. Nebeling in 1995 for two pediatric patients with astrocytoma [139]. The results of those two cases were very encouraging and the diet was described in detail in another publication [140]. Implementing a KD with additional calorie restriction in a female patient with glioblastoma multiforme clearly demonstrated that this intervention was able to stop tumor growth [132]. This was achieved, however, on the expense of a dramatic weigh loss of 20% over the intervention period, which is no option for the majority of metastatic cancer patients being in a catabolic state. A first clinical study applying a non-restricted KD for patients with glioblastoma (ERGO-study, NHI registration number NCT00575146), which was presented at the 2010 ASCO meeting [141], showed good feasibility and suggested some anti-tumor activity. The protocol of another clinical interventional trial (RECHARGE trial, NCT00444054) treating patients with metastatic cancer by a very low CHO diet was published in 2008 [142], and preliminary data from this study presented at the 2011 ASCO-meeting showed a clear correlation between disease stability or partial remission and high ketosis, independent of weight loss and unconscious caloric restriction of the patients [136]. While a randomized study for the treatment of prostate cancer patents applying the Atkins diet (NCT00932672) is currently recruiting patients at the Duke University, another trial posted at the clinical trials database (ClinicalTrials.gov) is not yet open for recruitment (NCT01092247). Very recently, two Phase I studies applying a ketogenic diet based on KetoCal^® ^4:1 started recruitment at the University of Iowa, intended to treat prostate cancer patients (KETOPAN, NCT01419483) and non-small cell lung cancer (KETOLUNG, NCT01419587). Thus, in the future, several data should be available to judge whether this kind of nutrition is useful as either a supportive or even therapeutic treatment option for cancer patients.

## Is there a role for carbohydrate restriction in the prevention of cancer?

"Prevention of cancer" can refer to either the inhibition of carcinogenesis *per se *or - once that cells made the transition to malignancy - the sufficient delay of tumor growth, so that it remains undetected and asymptomatic during a subject's lifespan. There is evidence that even modest CHO restriction may influence both of these mechanisms positively through various pathways. The IGF1R-IR pathway has already been discussed: once a potentially carcinogenic somatic mutation has occurred, the probability for carcinogenesis of a cell that is borderline between apoptosis and malignancy might be raised by high levels of insulin and IGF1 in the micro-environment. Once a cell became malignant, high insulin and IGF1 levels might accelerate proliferation and progression towards a more aggressive, glycolytic phenotype. In rats treated with the carcinogen N-methyl-N-nitrosourea, it has been shown that lowering the CHO content of the diet from 60 E% to 40 E% with a simultaneous increase in protein was sufficient to lower postprandial insulin levels as well as decrease the appearance rate of tumors from (18.2 ± 1.3)%/wk to (12.9 ± 1.4)%/wk (p < 0.05), however with no statistically significant effect on tumor latency and weight measured after 10 wk [143]. Similarly, a recent study reported that NOP mice, which normally have a 70 - 80% chance of developing breast cancer over their lifetime due to genetic mutations, stayed tumor-free at 1 year of age when their calories from CHO were limited to 15%, while almost half of those on a 55% CHO diet developed tumors [131]. Notably, only 3 out of 11 mice in the 15% CHO group died with having a tumor compared to 7 out of 10 in the 55% CHO group; at death, significantly lower plasma insulin levels had been measured for the low CHO group. These results support the epidemiological [25,29,31,32] and *in vitro *[81,144] findings that high CHO diets, in particular those including high GI foods, promote mammary tumorigenesis via the sustained action of insulin.

Lower insulin levels may further increase the chance of intermittent ketosis, in particular if CHO restriction is combined with exercise, calorie restriction or intermittent fasting. Seyfried and Shelton [40] pointed out the possibility of ketone bodies to help in cancer prevention through their ability to protect the mitochondria from inflammation and ROS. Being more satiating than low-fat diets [145,146], a low CHO diet would make it easier to avoid caloric overconsumption or to implement intermittent fasting as an additional lifestyle change [147].

### Avoidance of chronic inflammation

Another potential benefit of low CHO diets might lie in their influence upon inflammatory processes that take place within various tissues. Inflammation is a well-established driver of early tumorigenesis and accompanies most, if not all cancers [148]. Chronic, 'smouldering' inflammation can both cause and develop along with neoplasia. There is evidence that chronic intake of easily digestible CHOs is able to promote such an inflammatory state in leukocytes and endothelial cells [94]. In obese individuals [149] and healthy subjects who underwent eccentric exercise training [150], the inflammatory state was further augmented postprandially through a high CHO intake, but not through high-fat, low CHO meals in the latter study. Maybe more importantly, even moderate CHO restriction has been shown to effectively target several important markers of atherosclerosis and type II diabetes, both of which are associated with chronic inflammation [151-157]. Forsythe et al. showed that in overweight individuals with dyslipidemia a very low CHO diet had a more favorable effect than a low fat diet in reducing several markers of inflammation [158]. Given these findings, it can be hypothesized that a diet with a low GL positively affects cancer risk through reducing postprandial hyperglycemia and the associated inflammatory response.

In this context, it is important to note that a low CHO diet offers further possibilities to target inflammation through omission or inclusion of certain foods. Usually, CHO restriction is not only limited to avoiding sugar and other high-GI foods, but also to a reduced intake of grains. Grains can induce inflammation in susceptible individuals due to their content of omega-6 fatty acids, lectins and gluten [159,160]. In particular gluten might play a key role in the pathogenesis of auto-immune and inflammatory disorders and some malignant diseases. In the small intestine, gluten triggers the release of zonulin, a protein that regulates the tight junctions between epithelial cells and therefore intestinal, but also blood-brain barrier function. Recent evidence suggests that overstimulation of zonulin in susceptible individuals could dysregulate intercellular communication promoting tumorigenesis at specific organ sites [161].

Paleolithic-type diets, that by definition exclude grain products, have been shown to improve glycemic control and cardiovascular risk factors more effectively than typically recommended low-fat diets rich in whole grains [162]. These diets are not necessarily very low CHO diets, but focus on replacing high-GI modern foods with fruits and vegetables, in this way reducing the total GL. This brings us back to our initial perception of cancer as a disease of civilization that has been rare among hunter-gatherer societies until they adopted the Western lifestyle. Although there are certainly many factors contributing to this phenomenon, the evidence presented in this review suggests that reduction of the high CHO intake that accounts for typically > 50 E% in the Western diet may play its own important role in cancer prevention and outcome.

## Conclusions

We summarize our main findings from the literature regarding the role of dietary CHO restriction in cancer development and outcome.

(i) Most, if not all, tumor cells have a high demand on glucose compared to benign cells of the same tissue and conduct glycolysis even in the presence of oxygen (the Warburg effect). In addition, many cancer cells express insulin receptors (IRs) and show hyperactivation of the IGF1R-IR pathway. Evidence exists that chronically elevated blood glucose, insulin and IGF1 levels facilitate tumorigenesis and worsen the outcome in cancer patients.

(ii) The involvement of the glucose-insulin axis may also explain the association of the metabolic syndrome with an increased risk for several cancers. CHO restriction has already been shown to exert favorable effects in patients with the metabolic syndrome. Epidemiological and anthropological studies indicate that restricting dietary CHOs could be beneficial in decreasing cancer risk.

(iii) Many cancer patients, in particular those with advanced stages of the disease, exhibit altered whole-body metabolism marked by increased plasma levels of inflammatory molecules, impaired glycogen synthesis, increased proteolysis and increased fat utilization in muscle tissue, increased lipolysis in adipose tissue and increased gluconeogenesis by the liver. High fat, low CHO diets aim at accounting for these metabolic alterations. Studies conducted so far have shown that such diets are safe and likely beneficial, in particular for advanced stage cancer patients.

(iv) CHO restriction mimics the metabolic state of calorie restriction or - in the case of KDs - fasting. The beneficial effects of calorie restriction and fasting on cancer risk and progression are well established. CHO restriction thus opens the possibility to target the same underlying mechanisms without the side-effects of hunger and weight loss.

(v) Some laboratory studies indicate a direct anti-tumor potential of ketone bodies. During the past years, a multitude of mouse studies indeed proved anti-tumor effects of KDs for various tumor types, and a few case reports and pre-clinical studies obtained promising results in cancer patients as well. Several registered clinical trials are going to investigate the case for a KD as a supportive therapeutic option in oncology.

## List of abbreviations

AMPK: AMP-activated protein kinase; CHO: carbohydrate; CT: computed tomography; E%: percentage of energy; FDG: ^18^F-fluoro-2-deoxyD-glucose; GI: glycemic index; GL: glycemic load; HIF-1α: hypoxia inducible factor-1α; IGF: insulin like growth factor; IR: insulin receptor; KD: ketogenic diet; LCT: long chain triglycerides; MMP: matrix metalloproteinase; MCT: medium chain triglycerides; mTOR: mammalian target of rapamycin; PET: positron emission tomography; PI13K: Phosphoinositide 3-kinase; ROS: reactive oxygen species.

## Competing interests

The authors declare that they have no competing interests.

## Authors' contributions

RJK drafted the manuscript, UK drafted figures and parts of the manuscript, both authors finalized the manuscript. All authors have read and approved the final manuscript.
